# Sesamin inhibits IL-1β-stimulated inflammatory response in human osteoarthritis chondrocytes by activating Nrf2 signaling pathway

**DOI:** 10.18632/oncotarget.13360

**Published:** 2016-11-15

**Authors:** Pengyu Kong, Guanghua Chen, Anlong Jiang, Yufu Wang, Chengchao Song, Jinpeng Zhuang, Chunyang Xi, Guangxi Wang, Ye Ji, Jinglong Yan

**Affiliations:** ^1^ Department of Orthopedics Surgery, The Second Affiliated Hospital of Harbin Medical University, Harbin 150001, China

**Keywords:** sesamin, osteoarthritis chondrocyte, IL-1β, NF-κB, Nrf2

## Abstract

Sesamin, a bioactive component extracted from sesame, has been reported to exert anti-inflammatory and anti-oxidant effects. In this study, we evaluated the anti-inflammatory effects of sesamin on IL-1β-stimulated human osteoarthritis chondrocytes and investigated the possible mechanism. Results demonstrated that sesamin treatment significantly inhibited PGE2 and NO production induced by IL-1β. Sesamin inhibited MMP1, MMP3, and MMP13 production in IL-1β-stimulated chondrocytes. Sesamin also inhibited IL-1β-induced phosphorylation of NF-κB p65 and IκBa. Meanwhile, sesamin was found to up-regulate the expression of Nrf2 and HO-1. However, Nrf2 siRNA reversed the anti-inflammatory effects of sesamin. In conclusion, our results suggested that sesamin showed anti-inflammatory effects in IL-1β-stimulated chondrocytes by activating Nrf2 signaling pathway.

## INTRODUCTION

Osteoarthritis (OA) is a chronic articular disease characterized by degradation and destruction of cartilage matrix [1, 2]. It often affects joints and leads to intense pain in aged people [3]. Studies showed that inflammation are closely integrated processes in OA and may affect disease progression and pain [4]. Inflammatory cytokine network plays a critical role in the progression of OA [5]. IL-1β, an important cytokine in the progression of OA, could induce the production of matrix metalloproteinases (MMPs) and inflammatory mediator PGE_2_ and NO production in chondrocytes [6, 7]. These inflammatory mediators lead to the clinical manifestations of OA [8]. Accumulated evidences suggested that inhibition of IL-1β-induced inflammatory response may represent a useful strategy to treat OA [9]. Nrf2 has been reported to play important roles in the regulation of oxidative stress. Furthermore, activation of Nrf2 signaling pathway could inhibit NF-κB activation and inflammatory mediator production.

Sesamin, the main component of sesame seed and its oil, has been reported to have anti-inflammatory and anti-oxidative effects [10]. Sesamin has been reported to inhibit LPS-induced inflammation and extracellular matrix catabolism in rat intervertebral disc [11]. Sesamin has been reported to attenuate LPS-induced acute lung injury in mice [12]. Sesamin also has protective effects against LPS/D-galactosamine-induced liver injury in mice [13]. Furthermore, sesamin has been reported to inhibit LPS-induced proliferation and invasion in prostate cancer cells [14]. In addition, sesamin has been shown to inhibit HMGB1-induced vascular barrier disruptive responses [15]. However, there was no study have been reported to investigate the anti-inflammatory effects and mechanism of sesamin in IL-1β-stimulated chondrocytes. In the present study, we investigated the anti-inflammatory effect and mechanism of sesamin on IL-1β-stimulated human osteoarthritis chondrocytes.

## RESULTS

### Effects of sesamin on chondrocytes viability

**Figure 1:**
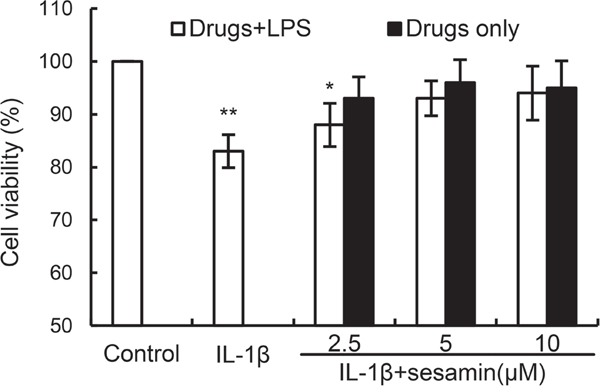
Effects of sesamin on the cell viability of chondrocytes The values presented are the means ± S.E.M. of three independent experiments. ^*^P < 0.05, **P < 0.01 *vs.* control group.

The effects of sesamin on the viability of chondrocytes were detected in this study. The results showed that IL-1β decreased the cell viability of chondrocytes. However, sesamin at concentration of 2.5 and 5μM reversed the effects of IL-1β on cell viability (Figure 1).

### Sesamin inhibits IL-1β-induced NO and PGE2 production

**Figure 2:**
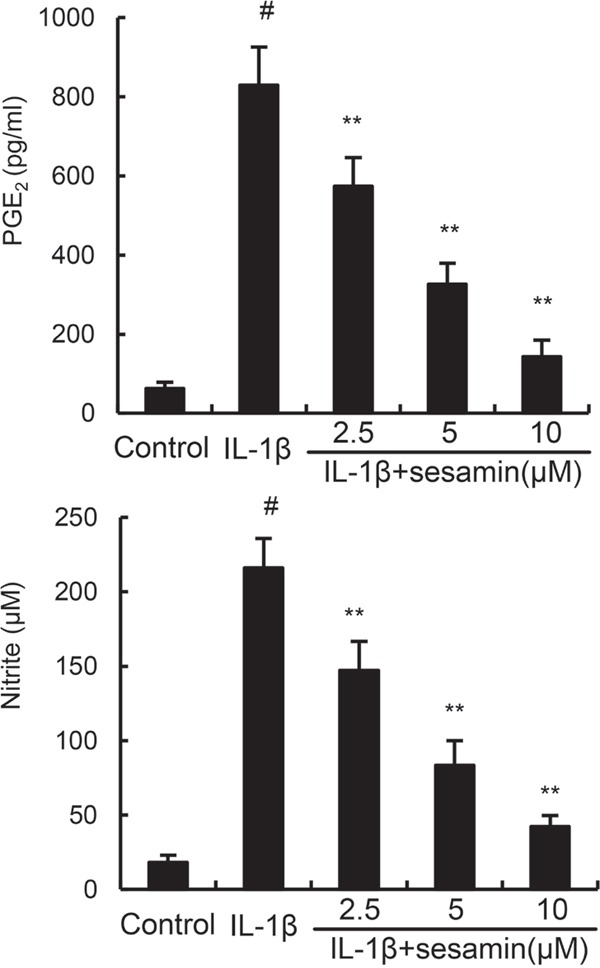
Sesamin inhibits IL-1β-induced NO and PGE_2_ production The data presented are the means ± S.E.M. of three independent experiments. ^#^*P* < 0.05 *vs.* control group; *P < 0.05, **P < 0.01 *vs.* IL-1β group.

Studies showed that inflammatory mediators play a critical role in inflammation. To investigate the anti-inflammatory effects of sesamin, the effects of sesamin on IL-1β-induced NO and PGE2 production were detected in this study. The results showed that IL-1β treatment obviously enhanced the levels of NO and PGE2 production. However, treatment of sesamin significantly reduced IL-1β-induced NO and PGE2 production (Figure 2).

### Sesamin inhibits IL-1β-induced MMP1, MMP3, and MMP13 production

**Figure 3:**
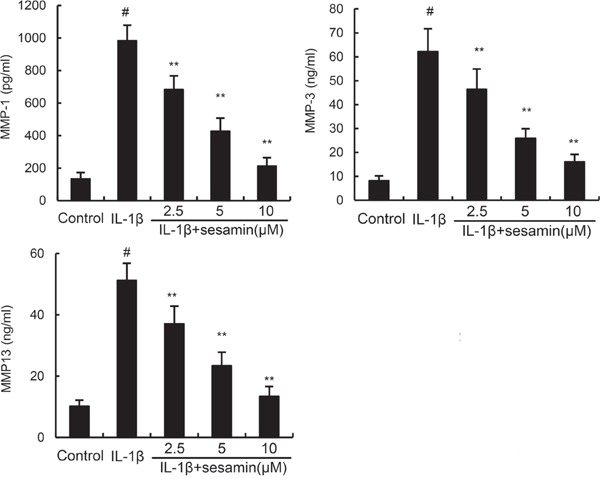
Sesamin inhibits IL-1β-induced MMP1, MMP3, and MMP13 production The data presented are the means ± S.E.M. of three independent experiments. ^#^*P* < 0.05 *vs.* control group; *P < 0.05, **P < 0.01 *vs.* IL-1β group.

In this study, the effects of sesamin on IL-1β-induced MMP1, MMP3, and MMP13 production were detected by ELISA. The results showed that IL-1β treatment obviously enhanced the levels of MMP1, MMP3, and MMP13 production. However, treatment of sesamin significantly reduced IL-1β-induced MMP1, MMP3, and MMP13 production (Figure 3).

### Sesamin inhibits IL-1β-induced NF-κB activation

**Figure 4:**
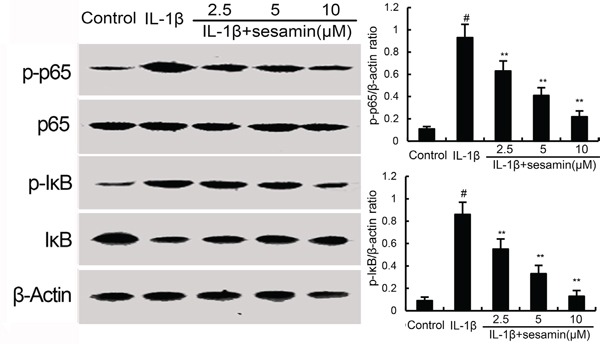
Sesamin inhibits IL-1β-induced NF-κB activation and IκBα degradation The values presented are the means ± S.E.M. of three independent experiments. ^#^*P* < 0.05 *vs.* control group; *P < 0.05, **P < 0.01 *vs.* IL-1β group.

NF-κB has been reported to play an important role in the regulation of inflammatory mediators production. To investigate the anti-inflammatory mechanism of sesamin, the effects of sesamin on IL-1β-induced NF-κB activation were measured by western blotting. The results showed that IL-1β significantly increased NF-κB phosphorylation and IκBα degradation. However, treatment of sesamin inhibited IL-1β-induced NF-κB activation in a dose-dependent manner (Figure 4).

### Effects of sesamin on Nrf2 and HO-1 expression

**Figure 5:**
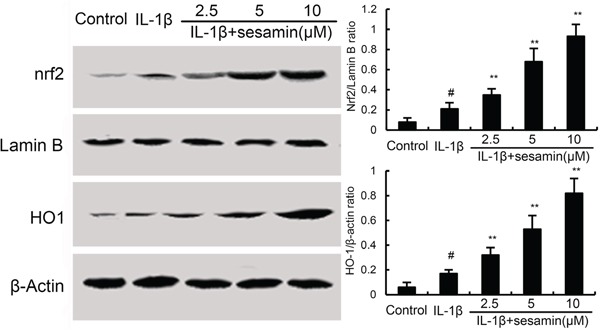
Effects of sesamin on Nrf2 signaling pathway The values presented are the means ± S.E.M. of three independent experiments. ^#^*P* < 0.05 *vs.* control group; *P < 0.05, **P < 0.01 *vs.* IL-1β group.

Several studies showed that activating of Nrf2 could inhibit inflammatory response. To further investigate the anti-inflammatory mechanism of sesamin, the effects of sesamin on Nrf2 signaling pathway were detected in this study. The results showed that IL-1β up-regulated the expression of Nrf2 and HO-1. And treatment of sesamin significantly up-regulated the expression of Nrf2 and HO-1 (Figure 5).

### Knockdown Nrf2 reversed the anti-inflammatory effects of sesamin

**Figure 6:**
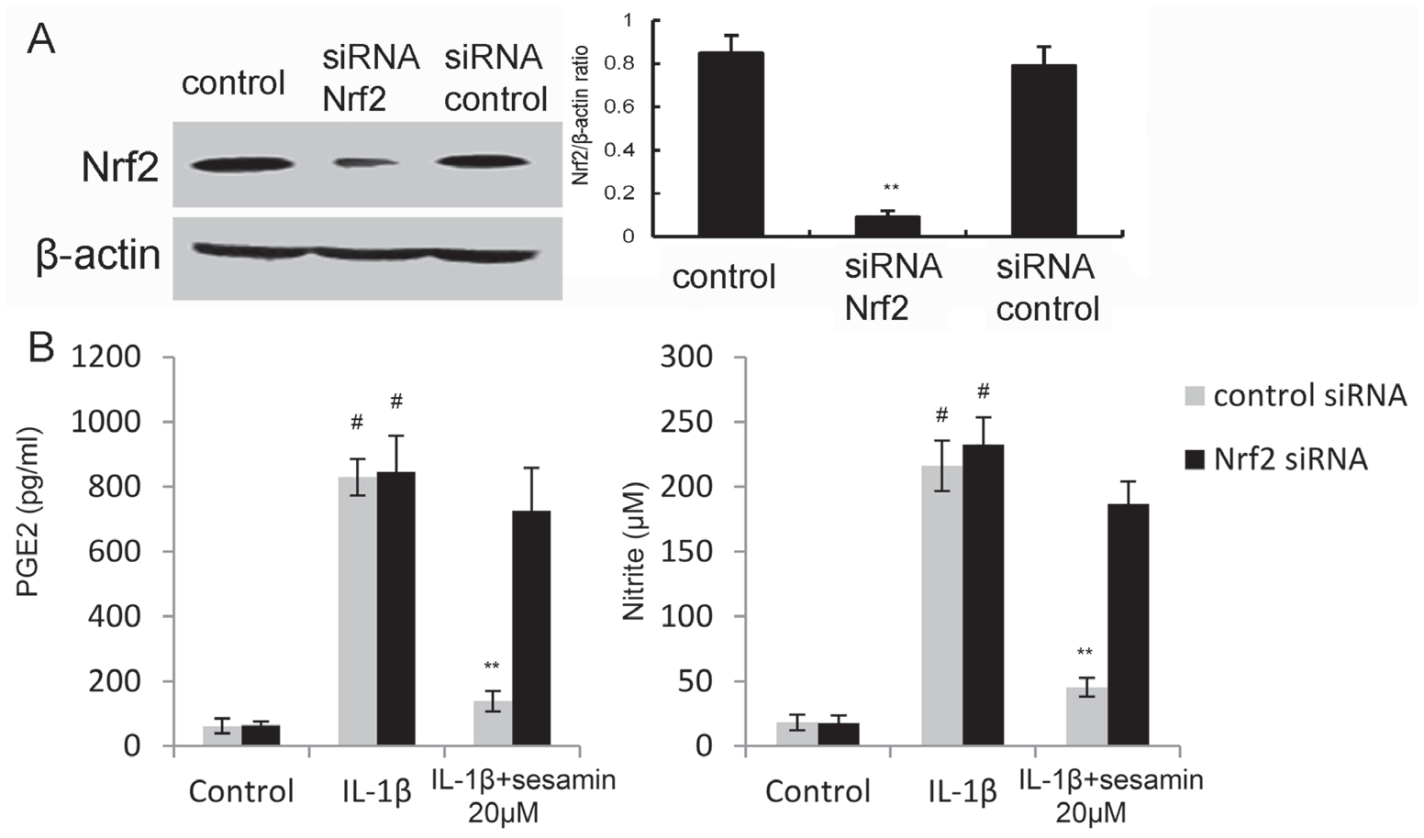
**A.** The effects of siRNA on Nrf2 expression was detected by Western blot analysis. **B.** Knockdown Nrf2 reversed the anti-inflammatory effects of sesamin. The values presented are the means ± S.E.M. of three independent experiments. ^#^*P* < 0.05 *vs.* control group; *P < 0.05, **P < 0.01 *vs.* IL-1β group.

To investigate whether the anti-inflammatory mechanism of sesamin was through Nrf2 signaling pathway, Nrf2 was knockdown by siRNA. The results showed that the expression of Nrf2 was inhibited after siRNA-Nrf2 transfection in chondrocytes (Figure 6A). In addition, the results showed that the inhibition of sesamin on PGE2 and NO production were reserved by Nrf2 siRNA (Figure 6B).

## DISCUSSION

In the present study, the results showed that sesamin dose-dependently inhibited IL-1β-induced PGE2, NO, MMP1, MMP3, and MMP13 production in chondrocytes. The results indicated that the anti-inflammatory property of sesamin was likely resulted from the inhibition of NF-κB activation through an Nrf2 dependent pathway.

A large number of studies showed that inflammation played a critical role in the development of OA by releasing a variety of inflammatory cytokines [16]. Among these cytokines, TNF-α and IL-1β played important roles [17]. Stimulating of chondrocytes with IL-1β could induce the production of MMPs and inflammatory mediators [18]. MMPs is an important risk factor that has the ability to inhibit type II collagen synthesis [19]. Among the MMPs, MMP1, MMP3, and MMP-13 could induce degradation of ECM in OA articular cartilage [20]. Inflammatory mediator PGE2 and NO also play critical roles in the development of OA [21]. PGE2 could attenuate extracellular matrix synthesis and NO could induce the release of MMPs and other inflammatory mediators [8]. Previous studies showed that inhibition the production of inflammatory mediators could attenuate the development of OA [21, 22]. In this study, our results showed that sesamin significantly inhibited IL-1β-induced PGE2 and NO, as well as MMP1, MMP3, and MMP13 production. These results indicated that sesamin could inhibit IL-1β-induced inflammatory response in chondrocytes.

NF-κB is a heterodimeric transcription factor composed of p50 and p65 subunits that expressed in many cell types [23, 24]. NF-κB can be activated by a variety of different stimuli and regulated a lot of inflammatory genes expression [25, 26]. In chondrocytes, IL-1β could induce NF-κB activation and inflammatory mediators, such as PGE2 and NO release [27]. To investigate the anti-inflammatory mechanism of sesamin, the effects of sesamin on IL-1β-induced NF-κB activation were detected in this study. Our results showed that sesamin significantly inhibited IL-1β-induced NF-κB activation. Nrf2, a critical transcription factor, is important for protecting cells against oxidative damage [28–30]. However, recent studies suggested that Nrf2 also has anti-inflammatory effect [31]. Activation of Nrf2/HO-1 signaling pathway could inhibit LPS-induced NF-κB activation [32]. To further investigate the anti-inflammatory mechanism of sesamin, the effects of sesamin on Nrf2 signaling pathway were measured. Our results showed that sesamin up-regulated the expression of Nrf2 and HO-1. Furthermore, the inhibition of sesamin on PGE2 and NO production were reserved by Nrf2 siRNA. These data suggested that the anti-inflammatory effects of sesamin were through activation of Nrf2/HO-1 signaling pathway.

In summary, our data demonstrate that sesamin has anti-inflammatory effects, as indicated by the inhibition of PGE2 and NO production. These effects are mediated by the inhibition of NF-κB activation through an Nrf2 dependent pathway. Sesamin may be a potential therapeutic agent for osteoarthritis.

## MATERIALS AND METHODS

### Chemicals and reagents

Sesamin (purity>98%) and MTT were purchased from Sigma-Aldrich (St. Louis, MO, USA). Recombinant human IL-1β was purchased from R&D systems (Minneapolis, MN, USA). Antibodies for Nrf2, HO-1, IκBα, and NF-κB were purchased from Santa Cruz Biotechnology (Santa Cruz, CA, USA). ELISA kits for MMP1, MMP3, MMP13 were purchased from R&D systems (Minneapolis, MN, USA). ELISA kit for PGE2 was purchased from eBioscience Inc (USA). Lipofectamine 2000 reagent was purchased from Invitrogen (Carlsbad, CA, USA).

### Cell culture

The experiment was in accordance with the Declaration of Helsinki and Tokyo. Articular cartilage samples were obtained from 12 patients (age: 54±8) undergoing total knee replacement surgery. Primary chondrocytes were isolated from articular cartilage as described previously [33]. The cells were cultured in DMEM containing 10% fetal bovine serum (FBS) and cultured at 37°C with 5% CO_2_. Cells between passages 1 to 3 were used in this study.

### MTT assay

Chondrocytes were seeded in a 96-well plate (10, 000 cells/well) and cultured overnight. Then, different concentrations of sesamin were added to each well and the cells were treated with IL-1β (10 ng/ml) for 24 h. Subsequently, MTT (5 mg/ml) was added to the cells and incubated for 4 h at 37°C. The medium was removed and the insoluble formazan product was dissolved in DMSO. Then, the optical density was measured at 450 nm on a microplate reader (TECAN, Austria).

### Inflammatory mediator assay

The levels of MMP1, MMP3, and MMP13 in cell culture supernatants were monitored by ELISA kits (R&D systems, Minneapolis, MN, USA). The concentration of PGE2 in cell culture supernatants was measured by an ELISA kit (eBioscience Inc, USA) according to the manufacturer's instructions. The concentration of NO in the culture medium was detected using the Griess reagent according to the manufacturer's instructions.

### Western blot analysis

Total proteins from chondrocytes were extracted using M-PER Mammalian Protein Extraction Reagent (Pierce, Rockford, IL). Protein concentration was determined using a Nanodrop 1000 spectrophotometer (Thermo, Wilmington, DE). The proteins were separated on 12% SDS-PAGE and transferred to PVDF membranes. After blocking, the membranes were incubated with Nrf2, HO-1, NF-κB p65, IκBα, p-IκBα antibodies at 4°C overnight. After washing three times, the membranes were incubated with HRP-conjugated IgG and detected by the ECL detection reagents (Thermo).

### Cells transfection with siRNA

Chondrocytes were seeded in a 6-well plate and cultured at 70% confluence. Then the cells were transfected with siRNA-Nrf2 and siRNA-scrambled with Lipofectamine 2000 according to the manufacturer's instructions. 24 h later, the cells were treated with sesamin and stimulated with IL-1β. The effects of siRNA on Nrf2 expression was detected by western blot analysis.

### Statistical analysis

Data are analyzed as the mean ± S.E.M. The differences between groups were evaluated by one-way analysis of variance followed by Dunnett's test. SPSS 11.5 software was used for all analysis. P <0.05 were considered to indicate statistical significance.
